# Development and validation of a novel questionnaire for self-determination of the range of motion of wrist and elbow

**DOI:** 10.1186/s12891-016-1171-z

**Published:** 2016-07-26

**Authors:** Marc Schnetzke, Svenja Schüler, Holger Keil, Sara Aytac, Stefan Studier-Fischer, Paul-Alfred Grützner, Thorsten Guehring

**Affiliations:** 1 Department for Trauma and Orthopaedic Surgery, BG Trauma Center Ludwigshafen at Heidelberg University Hospital, Ludwigshafen am Rhein, Germany; 2 Institute of Medical Biometry and Informatics, University of Heidelberg, Heidelberg, Germany; 3 Department for Trauma and Orthopaedic Surgery, BG Trauma Center Ludwigshafen at the University of Heidelberg, Ludwig Guttmann Strasse 13, 67071 Ludwigshafen am Rhein, Germany

**Keywords:** Self-assessment, Measurement tool, Validity, Reliability, Range of motion, Wrist, Elbow, Questionnaire

## Abstract

**Background:**

The aim of this study was to develop and validate a novel self-administered questionnaire for assessing the patient’s own range of motion (ROM) of the wrist and the elbow.

**Methods:**

In a prospective clinical study from January 2015 to June 2015, 101 consecutive patients were evaluated with a novel, self-administered, diagram-based, wrist motion assessment score (W-MAS) and elbow motion assessment score (E-MAS). The questionnaire was statistically evaluated for test-retest reliability, patient-physician agreement, comparison with healthy population, and influence of covariates (age, gender, affected side and involvement in workers’ compensation cases).

**Results:**

Assessment of patient-physician agreement demonstrated almost perfect agreement (k > 0.80) with regard to six out of eight items. There was substantial agreement with regard to two items: elbow extension (k = 0.76) and pronation (k = 0.75). The assessment of the test-retest reliability revealed at least substantial agreement (k = 0.70). The questionnaire revealed a high discriminative power when comparing the healthy population with the study group (*p* = 0.007 or lower for every item). Age, gender, affected side and involvement in workers’ compensation cases did not in general significantly influence the patient-physician agreement for the questionnaire.

**Conclusion:**

The W-MAS and E-MAS are valid and reliable self-administered questionnaires that provide a high level of patient-physician agreement for the assessments of wrist and elbow ROM.

**Level of evidence:** Diagnostic study, Level II

**Electronic supplementary material:**

The online version of this article (doi:10.1186/s12891-016-1171-z) contains supplementary material, which is available to authorized users.

## Background

Assessing the patient’s outcome and satisfaction is important in modern orthopedic practice [1–3]. Using questionnaires to evaluate patients with wrist and elbow disorders is widespread and has been shown to be valid and reproducible [4–9]. Self-reported outcome measures allow outcomes to be assessed from the patient’s perspective and do not require time in clinic or medical staff for data collection.

Common self-administered questionnaires for the determination of hand- and upper limp specific results of the wrist (e.g. patient-rated wrist evaluation, PRWE [8]) and of the elbow (e.g. The American Shoulder and Elbow Surgeons-Elbow, ASES-E [1]) enable the patient to assess the functional impairment of the joint, but they do not formally assess the range of motion, and patients have to attend clinic for this to be measured [10]. Therefore important data regarding the ROM would be lost in patients who are unable or unwilling to come to the outpatient clinic at the regular follow-up or for clinical research.

To our knowledge no validated self-assessment questionnaire for the ROM of the wrist or the elbow exists, which compares the agreement of the patient’s outcome with the examination by a physician.

Therefore, the aim of the current study was to develop a self-administered, diagram-based wrist motion assessment score (W-MAS) and elbow motion assessment score (E-MAS) to enable the patients to assess their own ROM of the wrist and the elbow. We further evaluated validity and reliability of this novel questionnaire with respect to the accuracy of self-determination of the wrist and elbow ROM.

## Methods

In this prospective, single-center study the novel questionnaire was evaluated in patients with elbow or wrist disorders. Before development of this novel questionnaire a PubMed search was performed to identify the currently available self-administered questionnaires for the wrist and elbow joints.

PubMed was searched in May 2016 for elbow- and wrist-specific MeSH-terms (‘elbow’ or ‘wrist’ in combination with ‘scoring system’, ‘outcome assessment’, ‘elbow disorder’, ‘wrist disorder’, ‘questionnaire’, ‘instrument’ and ‘clinical evaluation’), with no limit regarding the year of publication.

The PubMed search revealed 19 available self-administered questionnaires for the wrist- and elbow-joint, which are listed in Table 1. These questionnaires were analyzed regarding the assessment and validation method for the ROM. Most questionnaires combine self-assessment of subjective criteria and measurement of objective criteria (ROM) by a physician.

**Table 1:** Patient-administered questionnaires for the wrist and the elbow

Questionnaire	Joint	Self-assessment of ROM	Patient-physician validated ROM	Literature
Disabilities of the Arm, Shoulder and Hand (DASH)	Upper limb	No		Beaton et al. [5]
Patient-Rated Elbow Evaluation (PREE)	Elbow	No		MacDermid et al. [22]
American Shoulder and Elbow Surgeons-Elbow Score (ASES-E)	Elbow	No		King et al. [1]
Broberg and Morrey rating system (BMS)	Elbow	No		Broberg et al. [23]
Elbow Self-Assessment Score (ESAS)	Elbow	Yes	No	Beirer et al. [6]
Oxford Elbow Score (OES)	Elbow	No		Dawson et al. [24]
Liverpool Elbow Score (LES)	Elbow	No		Sathyamoorthy et al. [16]
Patient-Rated Tennis Elbow Evaluation Questionnaire (PRTEE)	Elbow	No		Vincent et al. [25]
Ewald scoring system	Elbow	No		Ewald [26]
Khalfayan score	Elbow	No		Khalfayan et al. [27]
Patient-Rated Wrist Evaluation (PRWE)	Wrist	No		MacDermid et al. [28]
Michigan Hand Outcomes Questionnaire (MHOQ)	Wrist	No		Chung et al. [29]
Musculoskeletal Functional Assessment (MFA)	Upper limb	No		Engelberg et al. [30]
Modern Activity Subjective Survey of 2007 (MASS07)	Wrist	No		Alexander et al. [4]
Levine Questionnaire (LQ)	Wrist	No		Levine et al. [31]
Mayo Wrist Score	Wrist	Yes	No	Cooney et al. [32]
Cooney and Bussey Score	Wrist	Yes	No	Cooney et al. [33]
Adelaide Questionnaire	Wrist	No		Bialocerkowski [34]
Munich Wrist Questionnaire	Wrist	Yes	No	Beirer et al. [35]

### Questionnaire development

A literature search was performed to determine the standard ROM of the elbow, the proximal and distal radioulnar joint (PRUJ and DRUJ) and the wrist (Table 2). Based on these data, a diagram-based questionnaire was developed.

**Table 2:** Mean ROM of the elbow and the wrist regarding literature

Joint	Movement	Range	Literature
Elbow	Extension-Flexion	0-0-140	Lockard et al. [36]
PRUJ/DRUJ	Supination-Pronation	90-0-70	Lockard et al. [36]
Wrist	Extension-Flexion	70-0-80	Ryu et al. [37]
Wrist	Radial and Ulnar deviation	30-0-20	Ryu et al. [37]

The extension and flexion of the elbow and the radial and ulnar deviation of the wrist were imaged with increments of 10°. Forearm supination and pronation as well as the extension and flexion of the wrist were imaged with an increment of 20°. These increments have been chosen to improve reproducibility without loss of information. For the assessment of the ROM, photographs were taken of a volunteer with an unimpaired wrist and elbow function. The joint position was measured and the volunteer was asked to hold the position briefly for the photograph. Additionally, the correct joint angle was then controlled with an electronic measurement device of Microsoft Word™ to ensure an accurate joint position on each image (Additional file 1: E-MAS, Additional file 2: W-MAS).

Finally, the E-MAS consists of 15 images for the extension (seven items, E-Ext) and flexion (eight items, E-Flex) of the elbow and ten images for supination (six items, Sup) and pronation (five items, Pro) of the forearm. The W-MAS consists of nine images addressing the extension (five items, W-Ext) and flexion (four items, W-Flex) of the wrist and six images addressing the radial- (three items, Rad) and ulnar deviation (four items, Uln).

Supination and pronation was performed with 90° of elbow flexion and the upper arm adjacent to the torso. To standardize forearm supination and pronation without rotation of the shoulder a short instructive direction was set in front of the related images. The objectivity of application and data interpretation (content validity) is guaranteed by the questionnaire-format with a clear answer form (possible-not possible) [11, 12].

### Questionnaire administration

Prior to a physical examination, the patient was asked to fill out the questionnaire. According to the directions on the questionnaire, patients were asked to check whether they are able to achieve the movements on each photograph for the wrist and the elbow on both sides. After completion of the questionnaire, the actual elbow and wrist ROM of both sides was measured by a single examiner [MS] using a standard 12-inch goniometer, regardless of whether the wrist and/or the elbow were the affected joint. The examiner was blinded to the self-reported ROM.

For assessment of the test-retest reliability the questionnaire was sent to the patients after an average of 65 (SD 21) days after the visit to clinic to prevent recall bias. Additionally, the patients were asked if there had been a change in the motion of any involved joint, and they were excluded from the assessment of test re-test-reliability in the case of a positive answer.

### Study population

Between January 2015 and July 2015, 101 consecutive patients suffering from disorders of the wrist and/or elbow were recruited at our outpatient clinic for participation in this study. These patients were identified from a consecutive list of patients scheduled for a follow-up visit as a prospective cohort. The inclusion criteria were age over 18 and disorder of the wrist and/or the elbow on one side. A subset of 58 patients (those, who denied having an altered ROM at the time of re-test) was re-assessed for evaluating the test-retest reliability.

Another 30 healthy people were recruited as a control group to test if the questionnaire can distinguish between patients with and without disorders of the wrist or elbow. The inclusion criteria for the control group were age over 18 and unimpaired function of the upper extremity. Exclusion criteria for control group and the study group were cognitive diseases, psychiatric diseases, communication problems or dyslexia.

### Statistical analysis

Descriptive statistics (mean, standard deviation, median, interquartile range, absolute and relative frequencies) were calculated to characterize the study population. The W-MAS and E-MAS were validated according to the proposed quality criteria for measurement properties of health status questionnaires of Terwee et al. [12]. The statistical evaluation included the assessment of (1) test–retest reliability, (2) agreement analysis (criterion validity and construct validity with Spearman correlation of at least 0.7), (3) comparison with healthy population (responsiveness) and (4) influence of covariates (age, gender, affected side and involvement in workers’ compensation cases). Floor and ceiling effects and interpretability were considered to be not relevant for the validation of these questionnaires as no mean score can be achieved.

The self-assessments by patients and the assessments by physicians are both represented by ordinal variables (ROM categorized into 4 to 8 groups). In the case of a missing item, only this specific motion pattern could not be evaluated.

#### Test-retest reliability

The test-retest reliability defines the degree of agreement of repeated measurements in the same subjects measured to assess the repeatability and reproducibility of an instrument. The time period between the measurements should be long enough to avoid learning and memory effects, but at the same time short enough to ensure consistency of the clinical symptoms [12]. Our target was to perform retests 2 months after baseline assessment. According to literature the time interval should be at least 2 weeks to prevent recall bias [12].

Reliability between the repeated measurements was assessed using Spearman’s rank correlation and Kendall’s Tau b correlation coefficient. To assess the agreement, Cohen’s kappa and the probability of exact agreement (number of test-retest agreements/n, expressed as percentage) were derived.

#### Agreement analysis

An agreement analysis assesses the extent to which a new instrument relates to the true value or to a gold standard value. In the context of assessing the ROM, industrial robotic devices and optical motion analysis can be considered as the gold standard [13, 14]. For practical reasons physician ratings were chosen as the reference in the current study. The Spearman rank correlation was used to evaluate the relation between patients’ and physicians’ response. A positive rating for the agreement analysis was assumed when the Spearman correlation was at least 0.7 [12]. To assess the agreement, the probability of exact and approximate agreement (expressed as percentage) and Cohen’s kappa were calculated.

To investigate the agreement dependent on the severity of the disorder, we calculated rates of exact agreement and approximate agreement in three different categories (no/mild, moderate and severe disorder, Table 3). The classification of the severity of the disorders was adopted from the previous elbow and wrist specific sores [15, 16].

**Table 3:** Classification of three subgroups of severity of disorder for every item of the E-MAS and the W-MAS

	No/mild	Moderate	Severe
Elbow (in degrees)			
extension	0–10	20–30	40–60
flexion	130–140	110–120	70–100
supination	90	50–70	0–30
pronation	70	50	0–30
Wrist (in degrees)			
extension	70	60	0–40
flexion	70	60	0–40
radial deviation	20	10	0
ulnar deviation	30	20	0–10

Exact agreement was defined as those cases in which physician and patient chose an identical response. Approximate agreement was defined as agreement within one grade, in a positive or negative direction.

#### Comparison with healthy population

To assess the extent to which the questionnaires could distinguish healthy persons from persons with wrist or elbow disorder, Mann-Whitney U-tests were performed to compare the scoring.

#### Influence of covariates

The influence of age (in decades), gender, affected side (dominant or non-dominant upper limb) and involvement in workers’ compensation cases on the patient-physician agreement was analyzed using univariable logistic regression.

For agreement measures of categorical data, the benchmarks as described by Landis and Koch where used: 0.00 to 0.20, 0.21 to 0.40, 0.41 to 0.60, 0.61 to 0.80, and 0.81 to 1.00 indicate poor, fair, moderate, substantial, and almost perfect agreement, respectively [17]. The agreement should be at least 0.70 to be adequate [12].

Benchmarks for the Spearman rank correlation are not consistent in the literature. We used 0.00 to 0.20, 0.21 to 0.50, 0.51 to 0.80, and 0.81 to 1.0 indicating no, weak, moderate, and strong relationship, respectively.

A *p*-value < 0.05 is considered as statistically significant in a descriptive manner. Statistical analysis was performed using SPSS (version 21.0 for Windows).

## Results

### Study population

Mean age of the study group was 54.9 years (range 20 to 84, SD 13.6), 57 (56.4 %) were male and 44 (43.6 %) were female. In 55 patients (54.5 %) the right side and in 46 patients (45.5 %) the left side was affected. The dominant side was affected in 58 patients (57.4 %). Table 4 summarizes patient’s diagnosis, representing a wide spectrum of traumatic and degenerative elbow and/or wrist disorders. Forty-one patients (42.6 %) were involved in workers’ compensation cases. Assessment of the ROM of all patients by the physician revealed no significant difference in restriction of ROM between the proportion of elderly (≥54.2 years, median) and younger patients (Additional file 3).

**Table 4:** Distribution of injury pattern

Diagnosis	No. patients (%)
Complex elbow dislocation	27 (26.7)
Radial head fracture	19 (18.8)
Distal radius fracture	18 (17.8)
Simple elbow dislocation	8 (7.9)
Distal humerus fracture	6 (5.9)
Monteggia like lesion	5 (5.0)
Humeral shaft fracture	5 (5.0)
Proximal ulnar fracture	4 (4.0)
Essex-Lopresti-Injury	3 (3.0)
Forearm shaft fracture	2 (2.0)
Arthritis of the elbow	2 (2.0)
Osteochondritis dissecans Capitellum humeri	1 (1.0)
Loose bodies elbow	1(1.0)
Total	101 (100)

A subgroup of 58 patients (57.4 %) from the study population was evaluated for test-retest reliability. Mean age of this subgroup was 56.7 years (range 37 to 81, SD 11.7), 31 (53.4 %) were male and 27 (46.6 %) were female. Another 30 healthy participants were recruited as a control group, mean age was 24.9 years (range 20 to 61, SD 11.4), ten (33.3 %) were male and 20 (66.6 %) were female. None of the 131 patients found any of the given instructions difficult to understand or to follow. The questionnaire took a maximum of 5 min for patients to complete.

#### Test-retest reliability

The retest was performed in 58 patients at 65 days (range 21 to 112, SD 21) following baseline assessment. Results of test-retest reliability are shown in Table 5. The assessment of the test-retest reliability revealed at least substantial agreement (k = 0.693). Three items showed almost perfect agreement. Spearman correlation demonstrated moderate correlation for radial deviation of the wrist and strong correlation for the other seven items (0.849–0.998).

**Table 5:** Statistics for test-retest reliability for the different parts of the questionnaire, *n* = 58

*n* = 58	Kendall-Tau-b (95 %-CI)	Spearman’s rank correlation coefficient (95 %-CI)	Cohen’s kappa (95 %-CI)	Exact agreement (in %)
Elbow				
extension	0.87 (0.73–0.96)	0.90 (0.78–0.98)	0.78 (0.64–0.90)	82.8
flexion	0.91 (0.82–0.99)	0.94 (0.85–1.00)	0.81 (0.69–0.93)	86.2
supination	0.86 (0.76–0.94)	0.89 (0.78–0.96)	0.73 (0.56–0.87)	84.4
pronation	0.87 (0.73–0.96)	0.88 (0.76–0.97)	0.77 (0.60–0.91)	87.9
Wrist				
extension	0.98 (0.96–1.00)	1.00 (0.99–1.00)	0.88 (0.75–1.00)	94.8
flexion	0.99 (0.97–1.00)	1.00 (0.99–1.00)	0.91 (0.79–1.00)	96.5
radial deviation	0.76 (0.52–0.95)	0.76 (0.52–0.95)	0.76 (0.51–0.95)	91.4
ulnar deviation	0.82 (0.67–0.93)	0.85 (0.70–0.96)	0.69 (0.50–0.86)	84.5

#### Agreement analysis

Results on patient-physician agreement are shown in Table 6. Assessment of patient-physician correlation demonstrated almost perfect agreement (k > 0.80) with regard to six of the eight items. There was substantial agreement with regard to two items: elbow extension (k = 0.764) and pronation (k = 0.749). Spearman correlation was at least 0.882 indicating a strong correlation for all items. Exact agreement between the patient and the rating of the physician was found in 82.1 to 95 % dependent on the tested movement and they were in approximate agreement (within one value) in at least 98 % of cases.

**Table 6:** Statistics for patient-physician agreement for the different parts of the questionnaire, *n* = 101

*n* = 101	Kendall-Tau-b (95 %-CI)	Spearman’s rank correlation coefficient (95 %-CI)	Cohen’s kappa (95 %-CI)	Exact agreement (in %)
Elbow				
extension	0.91 (0.85–0.96)	0.95 (0.91–0.98)	0.76 (0.67–0.86)	82.1
flexion	0.95 (0.90–0.99)	0.97 (0.93–1.00)	0.86 (0.78–0.93)	90.1
supination	0.93 (0.85–0.99)	0.94 (0.87–1.00)	0.89 (0.81–0.96)	93.0
pronation	0.86 (0.76–0.94)	0.88 (0.78–0.96)	0.75 (0.62–0.86)	87.1
Wrist				
extension	0.95 (0.91–0.98)	0.97 (0.94–1.00)	0.83 (0.73–0.92)	90.1
flexion	0.92 (0.84–0.99)	0.93 (0.85–0.99)	0.91 (0.81–0.98)	95.0
radial deviation	0.89 (0.80–0.98)	0.90 (0.80–0.98)	0.87 (0.76–0.97)	95.0
ulnar deviation	0.87 (0.78–0.95)	0.89 (0.80–0.97)	0.80 (0.69–0.90)	89.1

In the case of disagreement between the physician and patient responses, patients tended to err toward underestimating their ROM, meaning that the patient rated the ROM worse than the physician did. In 2–12.9 % patients tended to err towards underestimating their ROM and in 1 to 5 % patients tended to err towards overestimating their ROM.

**Fig. 1:**
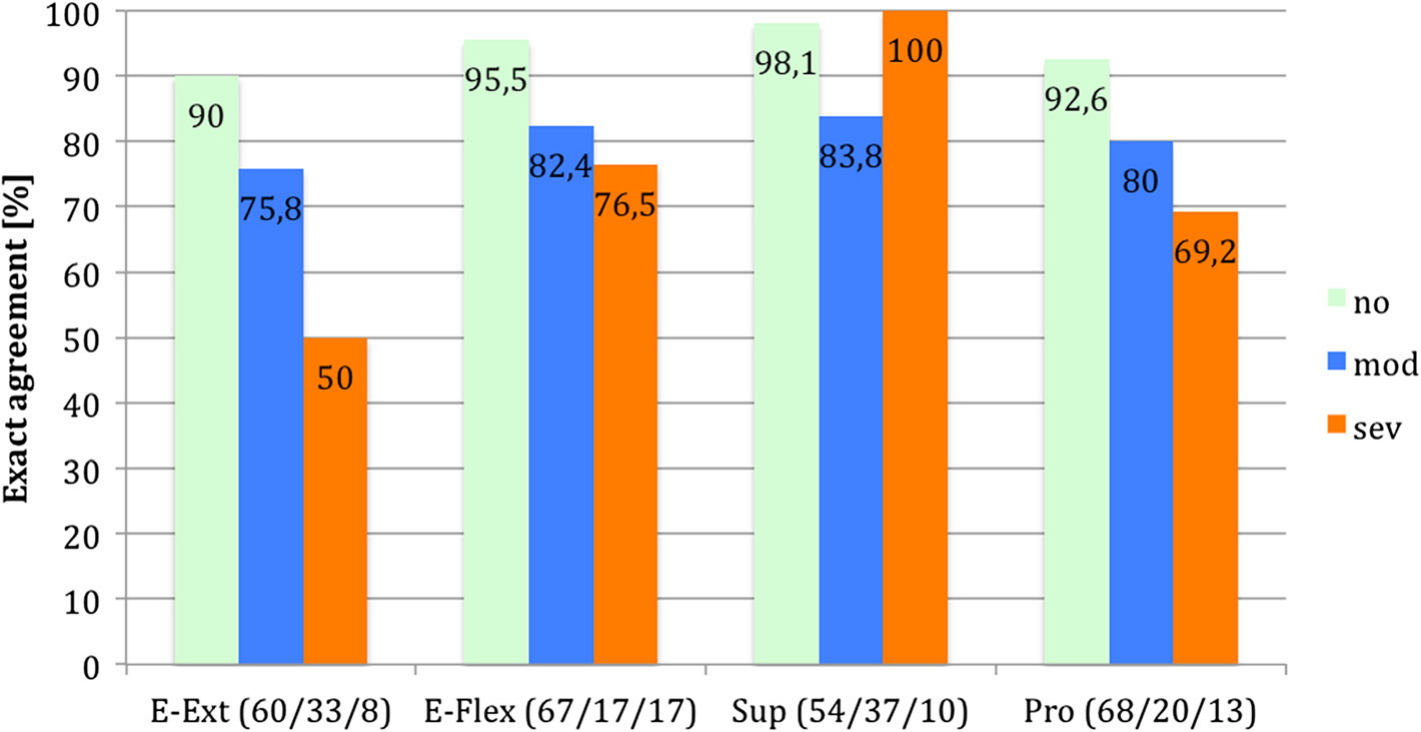
Percentage of exact agreement dependent on severity of disorder (E-MAS). The number of patients for each category is shown behind its respective item

**Fig. 2:**
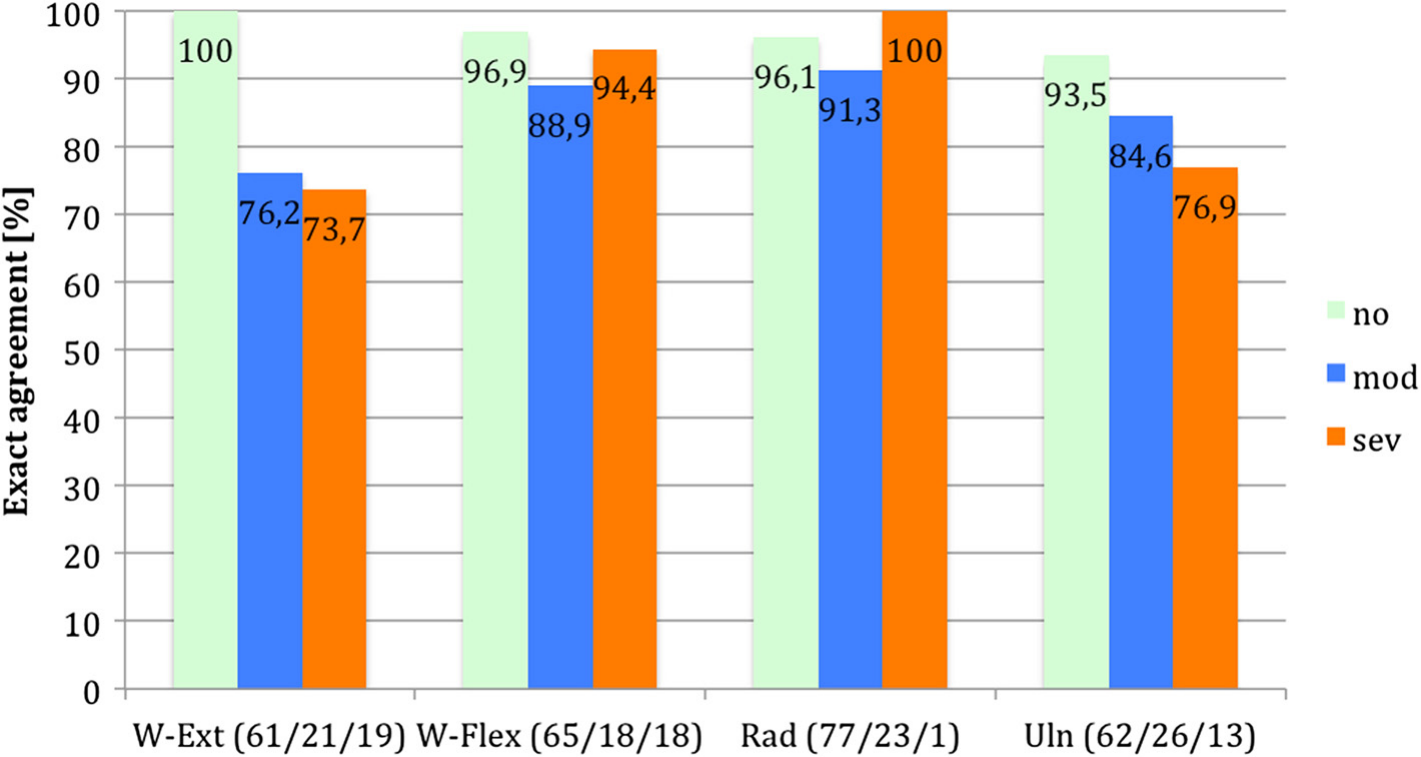
Percentage of exact agreement dependent on severity of disorder (W-MAS). The number of patients for each category is shown behind its respective item

The analysis of exact agreement dependent on the severity of the disorder demonstrated that patients tended to miss exact agreement with increasing severity of the disorder in five of eight items (Figs. 1 and 2). Patients with a severe restriction of the elbow extension (*n* = 8) showed an exact agreement in only 50 % of cases. In the other items exact agreement was found in at least 69.2 % in patients with a severe restriction of joint movement.

#### Comparison with healthy population

The comparison of the study population with the healthy control group demonstrated that the E-MAS and the W-MAS are able to distinguish between healthy people and patients with restriction of wrist or elbow joint motion (Table 7).

**Table 7:** Comparison of study population with healthy population for discriminative power for the different parts of the questionnaire

	Elbow disorder (*n* = 79)	Healthy population (*n* = 30)	*p*-value*
Elbow; median (IQR) [in degree]			
extension	10 (0–20)	0 (0)	<0.001
flexion	130 (110–140)	140 (0)	<0.001
supination	90 (70–90)	90 (0)	<0.001
pronation	70 (50–70)	70 (0)	0.001
	Wrist disorder (*n* = 22)	Healthy population (*n* = 30)	*p*-value*
Wrist; median (IQR) [in degree]			
extension	40 (40–70)	70 (70)	<0.001
flexion	40 (40–80)	80 (80)	<0.001
radial deviation	20 (17.5–20)	20 (20)	0.007
ulnar deviation	20 (10–30)	30 (30)	<0.001

*Mann-Whitney-U test

#### Influence of basic demographic data

Logistic regression analysis showed a significant effect of age on the ability to accurately assess elbow extension (*p* = 0.034). Younger patients were reliably able to identify elbow extension using the questionnaire, whereas elderly patients made significantly more errors. In all other items age did not affect the ability to achieve exact agreement with the questionnaire (*p* >0.05) and the OR was between 0.639 and 1.254.

No statistically significant association between ability to accurately assess wrist or elbow ROM and gender (*p* >0.05) or involvement of the dominant side (*p* >0.05) was observed.

Involvement with workers’ compensation cases significantly affected the ability to accurately assess the pronation (*p* = 0.039). All other items were not significantly influenced by the involvement with workers’ compensation (*p* >0.05).

Detailed analyses demonstrated that patients who were involved in workers’ compensation cases (*n* = 42, 41.6 %) tended to err towards underestimating their ROM in 27 of 43 cases (62.8 %).

## Discussion

In the present study the development and validation of a novel self-administered questionnaire for assessing the patient’s own range of motion of the wrist (W-MAS) and the elbow (E-MAS) are described. This study revealed substantial to almost perfect patient-physician agreement for the self-assessment of the wrist and elbow ROM. Evaluation of patient-physician correlation demonstrated almost perfect agreement (k >0.80) with regard to six of the eight items and two items (elbow extension and pronation) showed substantial agreement with k >0.70. In the case of mismatch between patient and physician, patients tended to err towards underestimating their ROM (53 of 78 cases; 68.9 %). In case of severe restriction of ROM patients tended to miss exact agreement in 23.5 to 50 % in five of eight items.

The assessment of the test–retest reliability was found to be almost perfect with Cohen’s kappa of k > 0.80 for all eight items. The questionnaire revealed a high discriminative power when the healthy population was compared with the study group (*p* = 0.007 or smaller for all items).

Logistic regression analysis suggested that increasing age impairs the ability to accurately assess elbow extension (*p* = 0.034). Involvement with workers’ compensation cases also appeared to influence the ability to accurately assess the pronation (*p* = 0.039).

For all other items, age and involvement in workers’ compensation cases did not significantly influence the validity of the questionnaire. Gender (male vs. female) and affected side (dominant vs. non-dominant) did also not significantly influence the validity of the questionnaire. The W-MAS and the E-MAS are the first questionnaires to validate the patient-physician agreement for ROM of the wrist and the elbow. According to the proposed quality criteria for measurement properties of health status questionnaires of Terwee et al. both questionnaires, the W-MAS and E-MAS are fully validated [12].

Smith et al. and Carter et al. examined the agreement between physician and patient-derived values for the shoulder ROM. They concluded, that patients are able to accurately assess their own active shoulder ROM with the help of a diagram-based questionnaire, which is comparable to the results of the current study [10, 18].

Given the number of available wrist and elbow questionnaires, is there a need for a novel self-administered score of the elbow and the wrist? In current medical care, self-administered questionnaires are useful for the assessment of patient care and for recording outcomes in research [2, 19]. Physicians can benefit from mail-in questionnaires or internet-based reporting sites and they can use such a tool to track a patient’s objective progress over time. In the same way, once a patient has been formally discharged from regular care, patient-administered questionnaires can be completed and returned via the postal service, e-mail, self-reporting website or even a smartphone app. This approach can facilitate long-term follow-up of postoperative patients for clinical trials by use of the objective criterion, ROM. According to our literature search, most available self-administered questionnaires for the wrist and the elbow do not assess ROM. Three questionnaires were identified with self-assessment of ROM, but these were not validated for patient-physician agreement. Therefore, important data regarding the ROM would be lost in patients who are unable or unwilling to come to the outpatient clinic at the regular follow-up.

Another possible advantage of self-administered questionnaires for determining ROM is the fact that the physician’s influence on the data obtained is minimized. Concerns and priorities of the surgeon may differ from those of the patient [20]. On the other hand, self-assessment of objective and subjective criteria may contain some bias [21]. In this study, basic demographic data like age, gender or involvement in workers’ compensation showed (except for 2 items) no significant influence on the validity and reliability of the questionnaire.

Self-administered questionnaires have some limitations. Patient-physician agreement in the assessments of the range of motion depends on the ability of the patient to understand the images given. The motion patterns given in this study are simple compared to other joints like the shoulder. All participants in the study and control group were asked about the feasibility of the questionnaire. None of the patients had any problems following the instructions and completing the questionnaire. In the current study, patients with communication problems or dyslexia were excluded, as they would not be able to provide useful information for the study.

There are certain weaknesses inherent to this study. The assessment of ROM by physician is usually represented by a continuous variable. In the current study, the assessments by physicians and the patients are both represented by ordinal variables due to practicability and comparability. As with any patient-reported outcomes survey, some patients are not able to complete the questionnaire. An inability to complete the questionnaire should alert clinicians that these patients might need to be carefully monitored between office visits or after formally discharge from care. In case of major restriction of ROM a higher percentage of disagreement between the physician and patient was found. Those patients also may require more specialized follow-up in certain settings.

This study is limited by the heterogeneity of the included injury patterns, as the majority of patients had restriction of ROM due to traumatic elbow disorders. Further study may be needed to have this questionnaire generalizable to other elbow and wrist complaints.

## Conclusions

This novel, patient-administered questionnaire provides a high level of patient-physician agreement for assessing the range of motion of the wrist (W-MAS) and the elbow (E-MAS). Based on the present data, both questionnaires are quick, simple to answer, and fully validated, and can be a helpful addition to subjective self-assessment questionnaires of the wrist and the elbow. In addition with other self-assessment scores of the upper limb, these questionnaires can provide helpful information regarding ROM of wrist and elbow to obtain higher follow-up rates.

## Abbreviations

ASES-E, The American Shoulder and Elbow Surgeons-Elbow; DRUJ, distal radioulnar joint; E-Ext, elbow extension; E-Flex, elbow flexion; E-MAS, elbow-motion assessment score; Pro, pronation; PRUJ, proximal radioulnar joint; PRWE, patient-rated wrist evaluation; Rad, radial deviation; ROM, range of motion; Sup, supination; Uln, ulnar deviationW-Ext, wrist extension; W-Flex, wrist flexion; W-MAS, wrist-motion assessment score

## Additional files

Additional file 1: Elbow Motion Assessment Score (E-MAS). (PDF 306 kb) Additional file 2: Wrist Motion Assessment Score (W-MAS). (PDF 560 kb) Additional file 3: Comparison of the range of motion of the proportion of elderly (≥54.2 years, median) and younger patients. (DOC 31 kb)
