# Supplementary material for: Development and validation of a novel questionnaire for self-determination of the range of motion of wrist and elbow
Source: BMC Musculoskelet Disord. 2016 Jul 26;17:312. doi: 10.1186/s12891-016-1171-z (PMC4960848; doi:10.1186/s12891-016-1171-z)
Supplement: Additional file 1: — Elbow Motion Assessment Score (E-MAS). (PDF 306 kb) [file 12891_2016_1171_MOESM1_ESM.pdf]

QUESTIONNAIRE RANGE OF MOTION ELBOW/FOREARM

Dear Patient,

On the following pages you will find some pictures designed to measure the extent of movement in your elbow and forearm. Based on the pictures, please answer as accurately as possible for **BOTH** elbows and forearms.

Thank you for your cooperation!

Patient data:

Name:

Date of birth:

Please tick as appropriate:

Elbow affected (or elbow currently being treated):

Right ☐

Left ☐

Are you:

Right-handed? ☐

Left-handed? ☐

QUESTIONNAIRE

ELBOW

Please tick under **<u>EACH</u>** picture for **<u>BOTH</u>** sides if you are able to perform the elbow movement as shown.

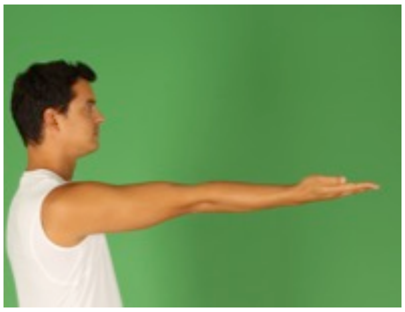

full extension, 0°

Right ☐

Left ☐

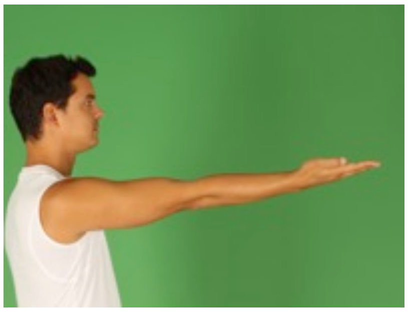

10° flexion

Right ☐

Left ☐

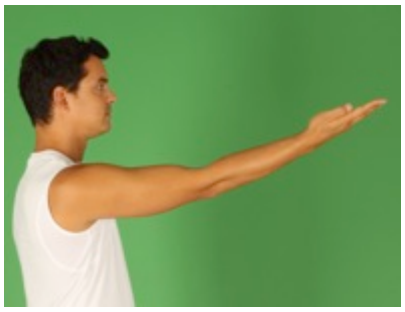

20° flexion

Right ☐

Left ☐

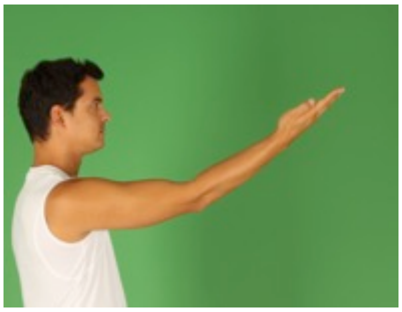

30° flexion

Right ☐

Left ☐

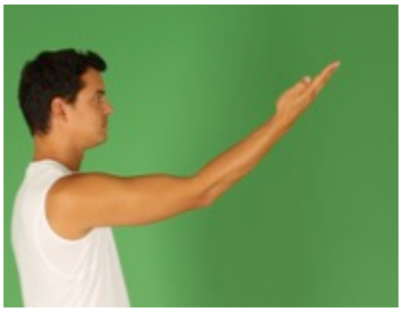

40° flexion

Right ☐

Left ☐

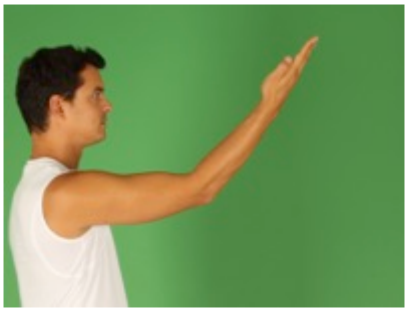

50° flexion

Right ☐

Left ☐

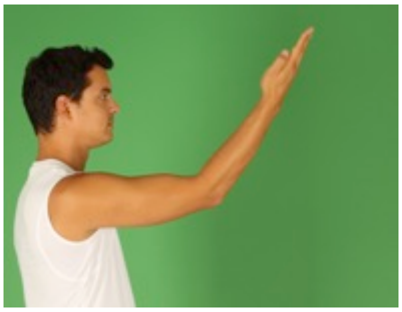

60° flexion

Right ☐

Left ☐

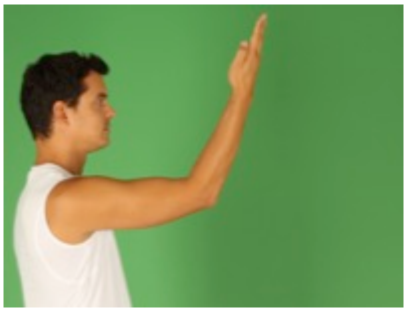

70° flexion

Right ☐

Left ☐

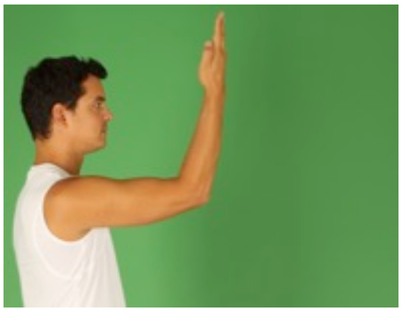

80° flexion

Right ☐

Left ☐

QUESTIONNAIRE

ELBOW

Please tick under **EACH** picture for **BOTH** sides if you are able to perform the elbow movement as shown.

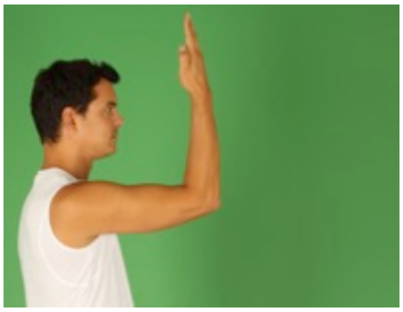

90° flexion
Right ☐
Left ☐

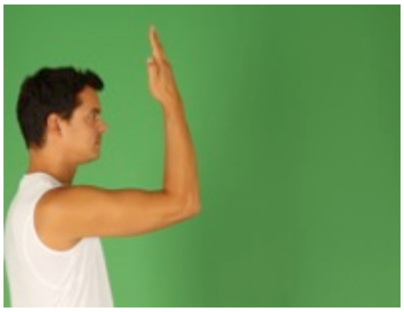

100° flexion
Right ☐
Left ☐

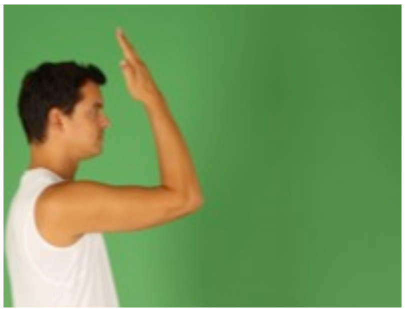

110° flexion
Right ☐
Left ☐

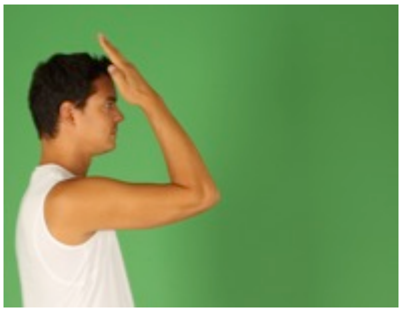

120° flexion
Right ☐
Left ☐

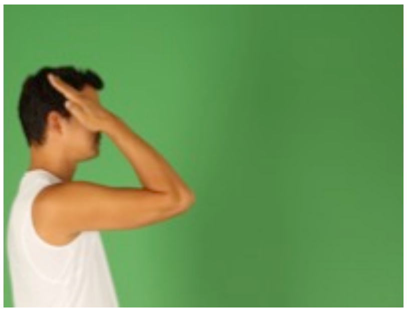

130° flexion
Right ☐
Left ☐

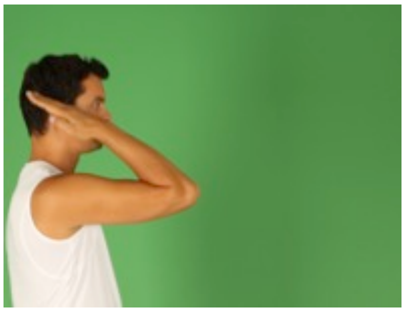

≥ 140° flexion
Right ☐
Left ☐

Comments:

QUESTIONNAIRE

FOREARM

Please tick under **EACH** picture for **BOTH** forearms if you are able to perform the movement as shown. Proceed as follows:
1) Hold your arm close to your side. 2) Bend your elbow at a right angle.

Outward and inward rotation of the forearm:

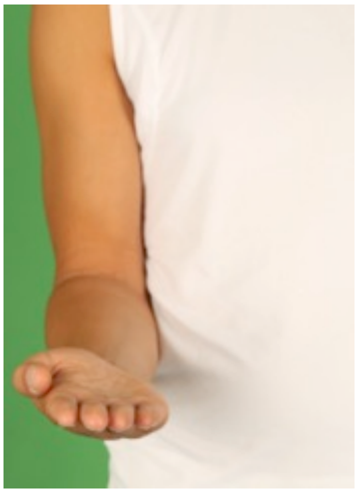

≥90° outward rotation
Right ☐
Left ☐

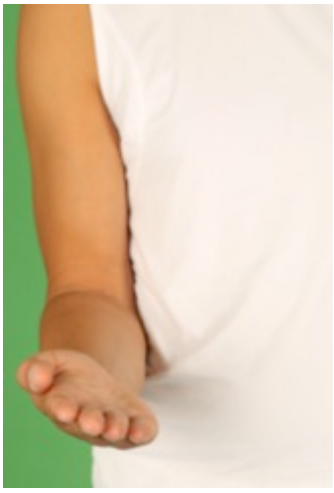

70° outward rotation
Right ☐
Left ☐

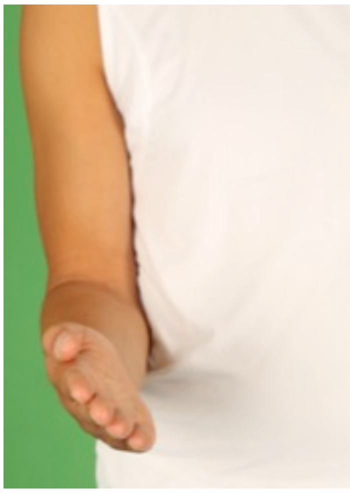

50° outward rotation
Right ☐
Left ☐

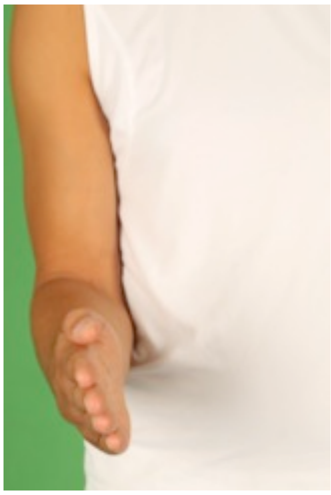

30° outward rotation
Right ☐
Left ☐

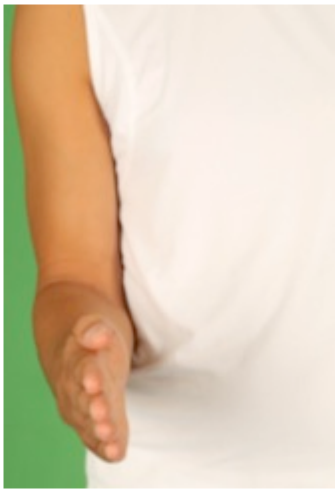

10° outward rotation
Right ☐
Left ☐

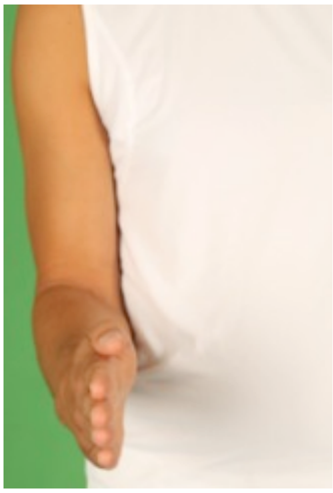

Straight, no rotation
Right ☐
Left ☐

QUESTIONNAIRE

FOREARM

Please tick under **EACH** picture for **BOTH** forearms if you are able to perform the movement as shown. Proceed as follows:
1) Hold your arm close to your side. 2) Bend your elbow at a right angle.

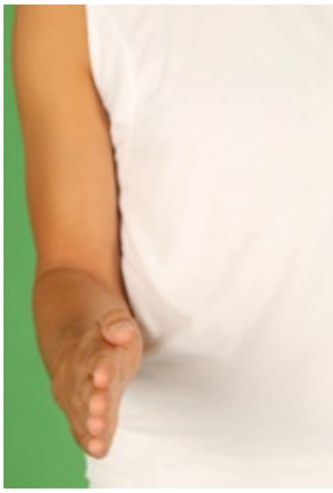

10° inward rotation
Right ☐
Left ☐

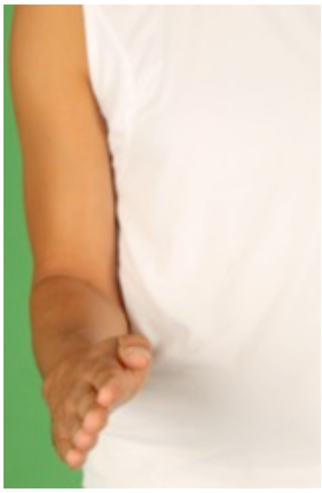

30° inward rotation
Right ☐
Left ☐

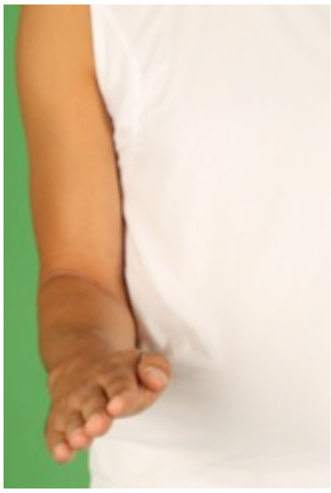

50° inward rotation
Right ☐
Left ☐

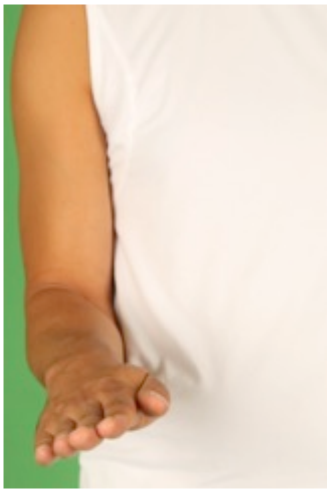

≥70° inward rotation
Right ☐
Left ☐

Comments:
