# Supplementary material for: Development and validation of a novel questionnaire for self-determination of the range of motion of wrist and elbow
Source: BMC Musculoskelet Disord. 2016 Jul 26;17:312. doi: 10.1186/s12891-016-1171-z (PMC4960848; doi:10.1186/s12891-016-1171-z)
Supplement: Additional file 2: — Wrist Motion Assessment Score (W-MAS). (PDF 560 kb) [file 12891_2016_1171_MOESM2_ESM.pdf]

QUESTIONNAIRE RANGE OF MOTION WRIST

Dear Patient,

On the following pages you will find some pictures designed to measure the extent of movement in your wrist. Please answer as accurately as possible for **BOTH** wrists.

Thank you for your cooperation!

Patient data:

Name:
Date of birth:

Please tick as appropriate:

Wrist affected (or wrist currently being treated):

Right ☐
Left ☐

Are you:

Right-handed? ☐
Left-handed? ☐

QUESTIONNAIRE

WRIST

Please tick under **EACH** picture for **BOTH** wrists if you are able to perform the movement as shown. To help, you can lay your hand on the edge of a table.

Extension and flexion of the wrist:

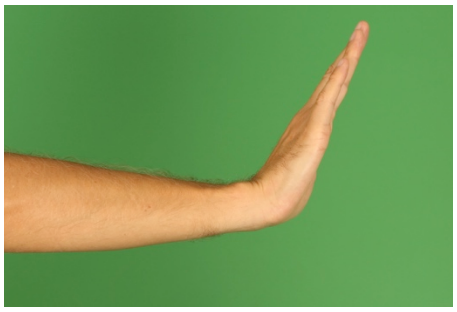

≥ 70° extension
Right ☐
Left ☐

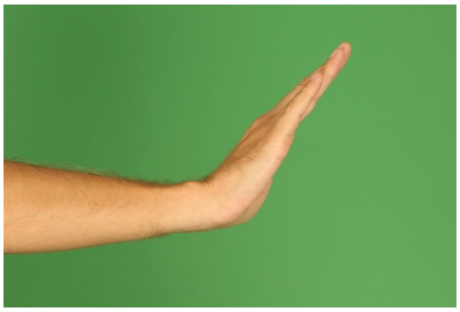

60° extension
Right ☐
Left ☐

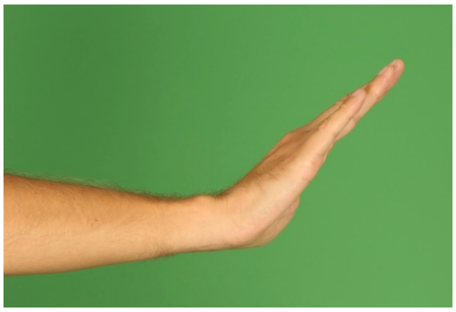

40° extension
Right ☐
Left ☐

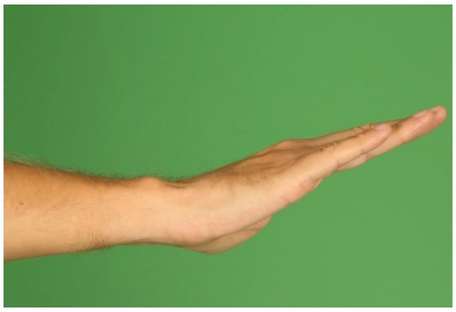

20° extension
Right ☐
Left ☐

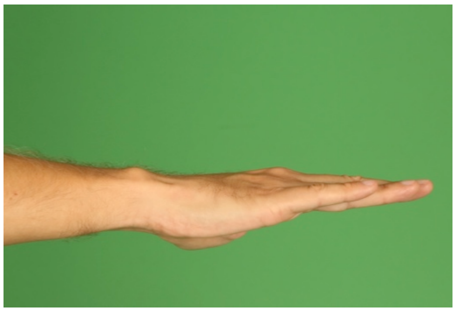

straight, 0°
Right ☐
Left ☐

QUESTIONNAIRE

WRIST

Please tick under **<u>EACH</u>** picture for **<u>BOTH</u>** wrists if you are able to perform the movement as shown. To help, you can lay your hand on the edge of a table.

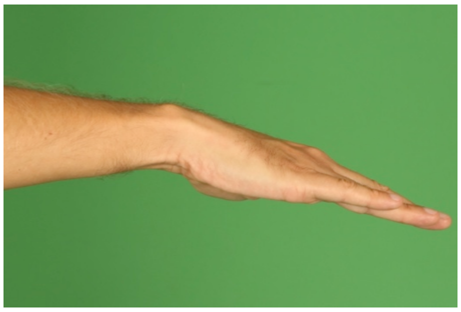

20° flexion

Right ☐

Left ☐

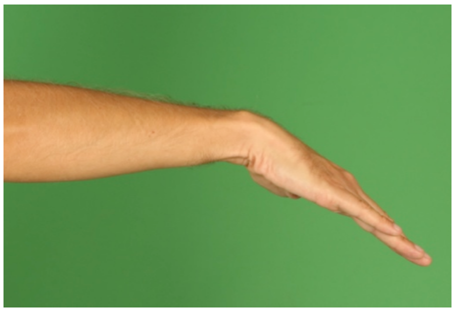

40° flexion

Right ☐

Left ☐

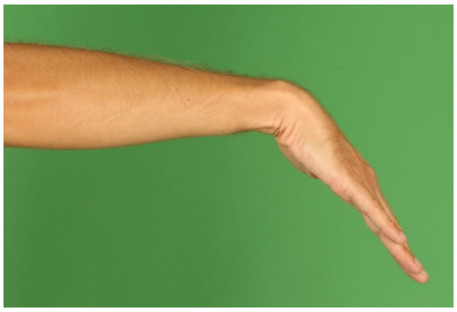

60° flexion

Right ☐

Left ☐

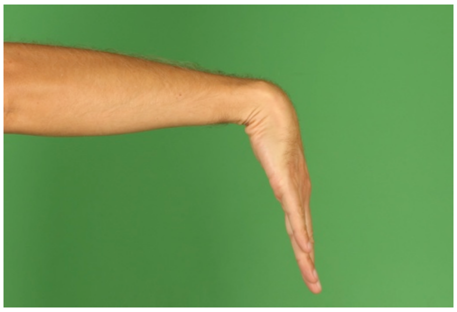

≥ 80° flexion

Right ☐

Left ☐

Comments:

QUESTIONNAIRE

WRIST

Please tick under **EACH** picture for **BOTH** wrists if you are able to perform the movement as shown. To help, you can lay your hand flat on a table.

Movement towards the thumb and little finger:

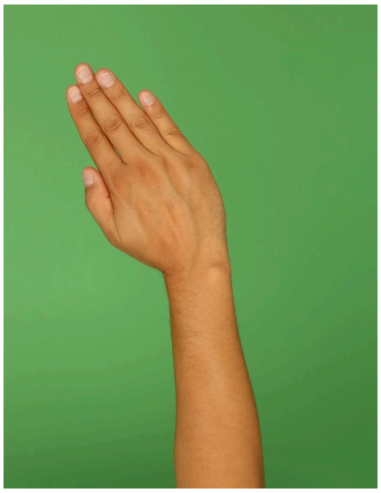

≥ 20° towards the thumb

Right ☐

Left ☐

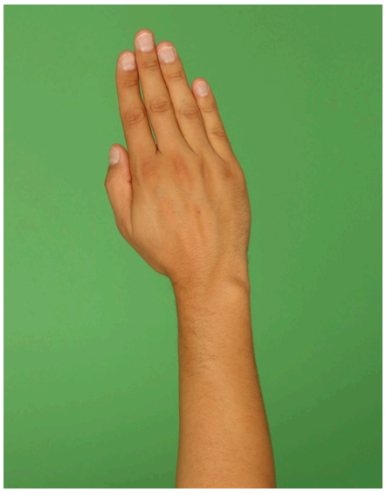

10° towards the thumb

Right ☐

Left ☐

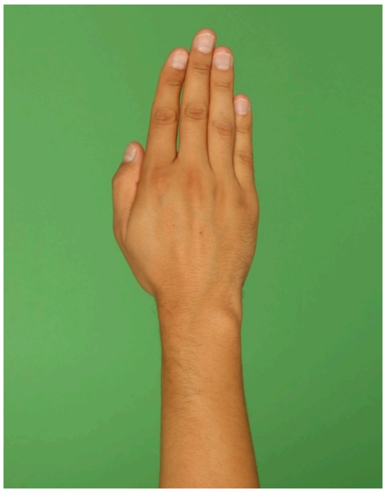

straight, 0°

Right ☐

Left ☐

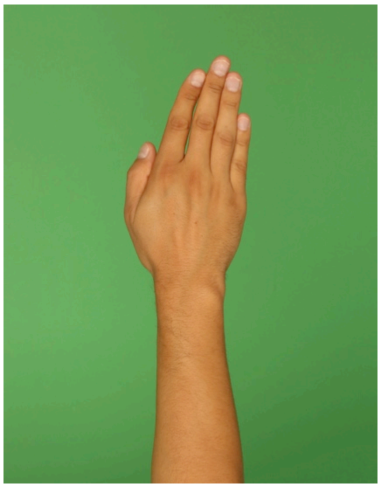

10° towards the little finger

Right ☐

Left ☐

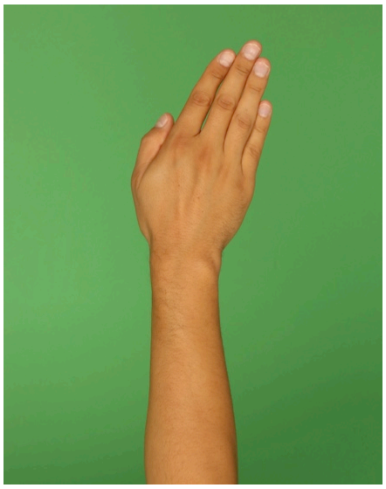

20° towards the little finger

Right ☐

Left ☐

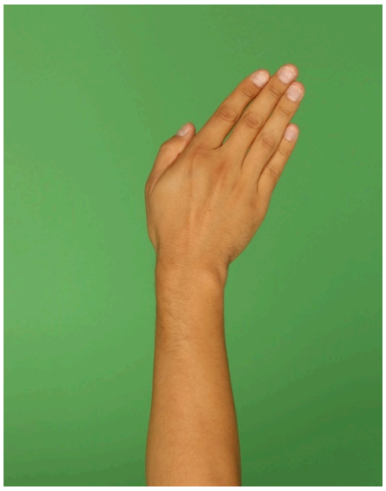

≥ 30° towards the little finger

Right ☐

Left ☐

Comments:
