# Supplementary material for: Development and validation of a novel questionnaire for self-determination of the range of motion of wrist and elbow
Source: BMC Musculoskelet Disord. 2016 Jul 26;17:312. doi: 10.1186/s12891-016-1171-z (PMC4960848; doi:10.1186/s12891-016-1171-z)
Supplement: Additional file 3: — Comparison of the range of motion of the proportion of elderly (≥54.2 years, median) and younger patients. (DOC 31 kb) [file 12891_2016_1171_MOESM3_ESM.doc]

|  | **All patients**  **(n=101)** | **Age ≥54.2**  **years**  **(n=51)** | **Age <54.2 years**  **(n=50)** | **p-value** |
| --- | --- | --- | --- | --- |
| Elbow, degree (SD)  extension  flexion  supination  pronation | **13.2 (SD 13.9)**  **125.8 (SD 18.5)**  **73.2 (SD 23.1)**  **60.0 (SD 16.6)** | **11.6 (SD 14.6)**  **128.2 (SD 17.9)**  **77.5 (SD 19.2)**  **62.0 (SD 14.5)** | **14.8 (SD 13.1)**  **123.3 (SD 18.9)**  **68.8 (SD 26.0)**  **58.0 (SD 18.5)** | **0.246**  **0.189**  **0.060**  **0.211** |
| Wrist, degree (SD)  extension  flexion  radial deviation  ulnar deviation | **63.5 (SD 9.5)**  **67.9 (SD 19.0)**  **17.5 (SD 4.6)**  **24.8 (SD 7.4)** | **64.3 (SD 9.2)**  **71.0 (SD 16.6)**  **18.2 (SD 3.9)**  **25.5 (SD 6.7)** | **62.6 (SD 9.9)**  **64.8 (SD 20.8)**  **16.8 (SD 5.1)**  **24.0 (SD 8.1)** | **0.369**  **0.102**  **0.114**  **0.316** |

Appendix C Assessment of the ROM of all patients by the physician
